# ACE2 Improves Right Ventricular Function in a Pressure Overload Model

**DOI:** 10.1371/journal.pone.0020828

**Published:** 2011-06-10

**Authors:** Jennifer A. Johnson, James West, Karen B. Maynard, Anna R. Hemnes

**Affiliations:** Division of Allergy, Pulmonary, and Critical Care Medicine, Vanderbilt University Medical Center, Nashville, Tennessee, United States of America; University of Florida, United States of America

## Abstract

**Background:**

Right ventricular (RV) dysfunction is a complication of pulmonary hypertension and portends a poor prognosis. Pharmacological therapies targeting RV function in pulmonary hypertension may reduce symptoms, improve hemodynamics, and potentially increase survival. We hypothesize that recombinant human angiotensin-converting enzyme 2 (rhACE2) will improve RV function in a pressure overload model.

**Results:**

rhACE2 administered at 1.8 mg/kg/day improved RV systolic and diastolic function in pulmonary artery banded mice as measured by *in vivo* hemodynamics. Specifically, rhACE2 increased RV ejection fraction and decreased RV end diastolic pressure and diastolic time constant (p<0.05). In addition, rhACE2 decreased RV hypertrophy as measured by RV/LV+S ratio (p<0.05). There were no significant negative effects of rhACE2 administration on LV function. rhACE2 had no significant effect on fibrosis as measured by trichrome staining and collagen1α1 expression. In pulmonary artery banded mice, rhACE2 increased Mas receptor expression and normalized connexin 37 expression.

**Conclusion:**

In a mouse RV load-stress model of early heart failure, rhACE2 diminished RV hypertrophy and improved RV systolic and diastolic function in association with a marker of intercellular communication. rhACE2 may be a novel treatment for RV failure.

## Introduction

Pulmonary hypertension (PH) is a broad term describing any elevation in mean pulmonary artery pressure greater than 25 mmHg at rest as determined by right heart catheterization. PH is caused by a variety of diseases including pulmonary arterial hypertension (PAH), PH secondary to left-sided heart disease, PH associated with lung disease and/or hypoxia, and PH resulting from chronic thrombotic/embolic disease [1]. Despite diverse etiologies, all categories of PH share right ventricular (RV) function as a critical determinant of morbidity and mortality [2], [3]. Importantly, RV dysfunction in PH can be reversible. For example, RV function improves after lung transplantation for PAH and after pulmonary endarterectomy in patients with chronic thromboembolic disease [4], [5]. Therefore, therapies focusing on RV function in PH may improve symptoms, quality of life, hemodynamics, and survival.

Pharmacological approaches limiting angiotensin II (Ang II) bioactivity (angiotensin-converting enzyme inhibitors and angiotensin receptor blockers) are the cornerstone of management of left ventricular (LV) dysfunction; however, there is no convincing evidence for use of these therapies in RV failure [6]. Inhibition of a hyperactive renin angiotensin system provides protection from LV remodeling, left heart failure, and mortality [7], [8], [9]. Recently, this class of therapeutics has expanded to include the novel enzyme, angiotensin-converting enzyme 2 (ACE2), which converts Ang II to Ang-(1-7). ACE2 is both present in the circulation and is an integral membrane protein in 72 organs including the heart [10], [11]. Conversion of Ang II to Ang-(1-7) by ACE2 has anti-hypertrophic, anti-proliferative, anti-fibrotic, and vasodilator properties in the LV [12], [13], [14], [15]. In various animal models of cardiac injury, ACE2 has been shown to be protective [15], [16], [17]. In an aortic banding model, recombinant human ACE2 (rhACE2) reversed LV hypertrophy, fibrosis, and improved diastolic dysfunction [18]. In human patients with left heart failure serum ACE2 is cardioprotective [19], [20].

Although the literature supports a beneficial role for ACE2 in LV function, the effects of ACE2 specifically on RV function have not been examined. Importantly, the response of the RV to stress should not be extrapolated from left heart experiments. The function, structure, and embryology of the right and left ventricles are unique. The RV is smaller, crescent shaped, thin-walled, and has a much lower afterload than the LV [21]; these differences are augmented by a differing embryologic origin of the RV [22], [23], [24], [25]. Thus, the RV may not respond similarly to the LV in response to stress and pharmacological therapies.

In preliminary studies, we demonstrated that ACE2 improves pulmonary vascular disease in a transgenic mouse model of PH, and now wish to study the effects of ACE2 on RV load-stress responses. We hypothesized that ACE2 would prevent RV hypertrophy and prevent hemodynamic dysfunction during RV load-stress. In order to study pharmaceutical effects of ACE2 on RV dysfunction in isolation, we administered rhACE2 to pulmonary artery banded (PAB) mice via osmotic pumps for two weeks. In this PAB model of early heart failure, we assessed structural, hemodynamic, and molecular effects of rhACE2 on the RV.

## Results

### rhACE2 decreases load-induced RV hypertrophy

**Figure 1:**
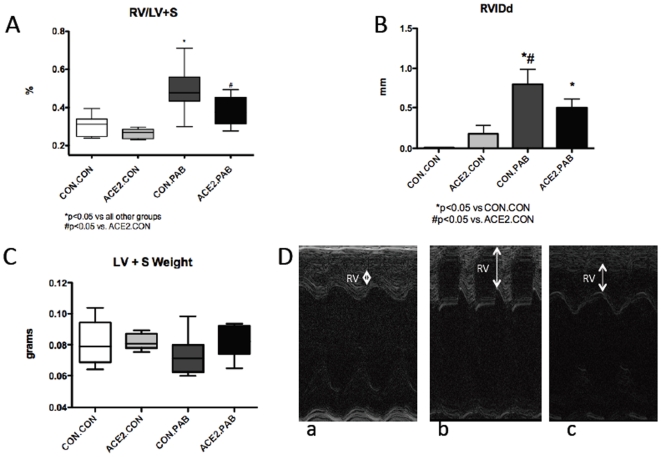
RV hypertrophy is decreased with recombinant human (rhACE2) administration. (A) RV/left ventricle (LV)+septum (S) ratio demonstrating RV hypertrophy in pulmonary artery banded (PAB) mice that is abrogated by rhACE2. (B) Right ventricular internal dimension in diastole (RVIDd) measured by M-mode echocardiography in PAB mice and controls with and without rhACE2. (C) There is no change in LV+S with rhACE2. (D) Representative M-mode echocardiography images with right ventricle labeled RV and RVIDd measured in (a) normal RV (b) CON.PAB and (c) ACE2.PAB.

PAB resulted in significant RV hypertrophy as measured by RV/LV+Septum (LV+S) ratio that was attenuated with rhACE2 administration (Figure 1). rhACE2 administration without load stress did not affect RV size. rhACE2 did not affect LV mass in control or PAB mice. Therefore, rhACE2 prevents load-induced RV hypertrophy, but has no effect on LV mass. In M-mode echocardiography, there was significant RV dilation in PAB mice with a trend (p = 0.32) towards decreased RV dilation with rhACE2 administration.

### rhACE2 improves RV systolic and diastolic function measured by in vivo hemodynamics

**Figure 2:**
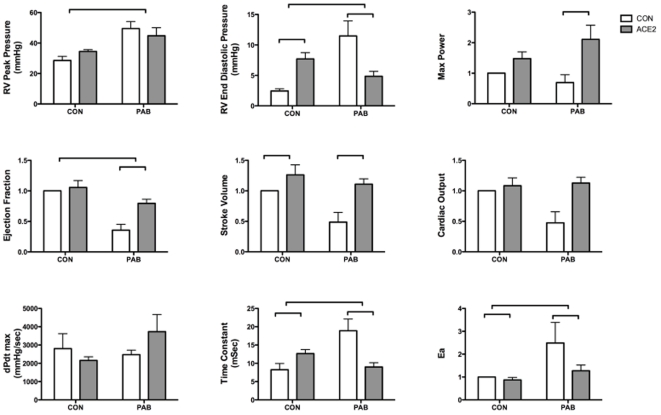
*In vivo* hemodynamic measurements in the RV. Long brackets denote effect of PAB p<0.05 by two-way ANOVA, short brackets denote effect of rhACE2 p<0.05 by two-way ANOVA. PAB decreased markers of systolic function (ejection fraction) and diastolic function (RV end diastolic pressure, diastolic time constant), and these changes were ameliorated by administration of rhACE2. dPdt max=maximum value of first derivative of ventricular pressure with respect to time during cardiac cycle. Ea=arterial elastance (end systolic pressure/stroke volume). Values formax power, ejection fraction, stoke volume, cardiac output, and Ea represent fold change from control.

The effects of 14 days of PAB or control with or without rhACE2 exposure on RV hemodynamics were studied (Figure 2). There were no differences in heart rate between groups (CON.CON 512±22 bpm, ACE2.CON 450±19 bpm, CON.PAB 475±37, ACE2.PAB 525±13). PAB resulted in increased RV peak pressure that is consistent with previously described PAB model results. PAB increased RV end diastolic pressure, diastolic time constant, and Ea (arterial elastance, end systolic pressure/stroke volume). Concomitantly, with PAB, there was a decrease in ejection fraction (EF). As expected with fixed banding of the pulmonary artery, administration of rhACE2 to PAB mice did not affect RV peak pressure. Administration of rhACE2 to control mice resulted in a small, but statistically significant increase in RV end diastolic pressure and stroke volume. In PAB mice, rhACE2 significantly decreased RV end diastolic pressure, increased maximal power, increased EF, increased stroke volume, decreased time constant, and decreased Ea by two-way ANOVA. In summary, in the context of PAB, there was improvement in both systolic and diastolic function with rhACE2.

### rhACE2 effect on LV function measured by in vivo hemodynamics

**Figure 3:**
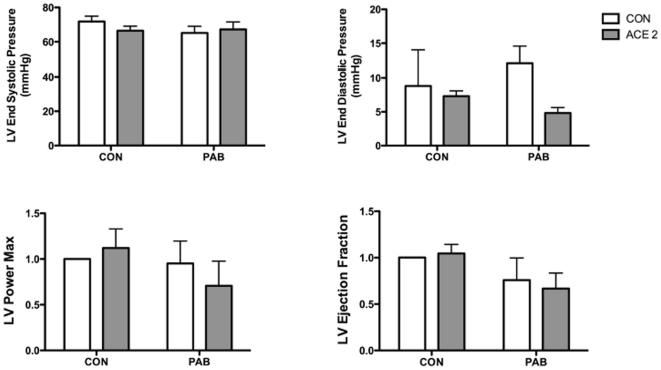
*In vivo* hemodynamic measurements in the LV. On two-way ANOVA, there were no significant findings, though there was a trend towards decreased LV end diastolic pressure (p = 0.08), LV power max, and LV ejection fraction with rhACE2 in PAB. Values represent fold change from control.

PAB had no significant effects on LV function in control animals as measured by *in vivo* hemodynamics (Figure 3). In PAB mice, there was a trend towards decreased LV end diastolic pressure with rhACE2 (p = 0.08) though this did not meet statistical significance.

### ACE2 is present in the RV

**Figure 4:**
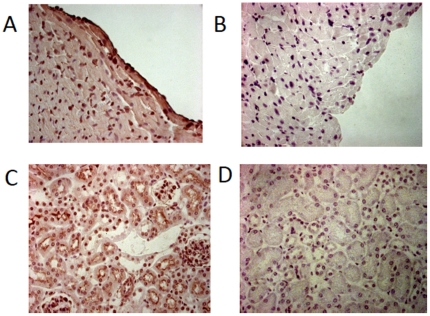
Immunohistochemistry for ACE2. (A) ACE2 stained RV, (B) control RV, (C) ACE2 stained kidney, (D) control kidney. 400× magnification.

ACE2 is an integral membrane protein present in the left atrium and LV, but to our knowledge the presence of ACE2 in right heart tissue has not been documented [10], [11]. To confirm the presence of ACE2 in the RV, we performed immunohistochemistry for ACE2 using kidney as positive control [10]. ACE2 stained in the myocardium, epicardium, and endocardium. In Figure 4, panel A demonstrates ACE2 staining of RV myocardium and endocardium. Panel C confirms ACE2 staining in kidney tissue [10]. Panel B and panel D depict RV and kidney control tissue not exposed to ACE2 antibody.

### rhACE2 did not diminish RV fibrosis or increase number of microvessels in PAB model

**Figure 5:**
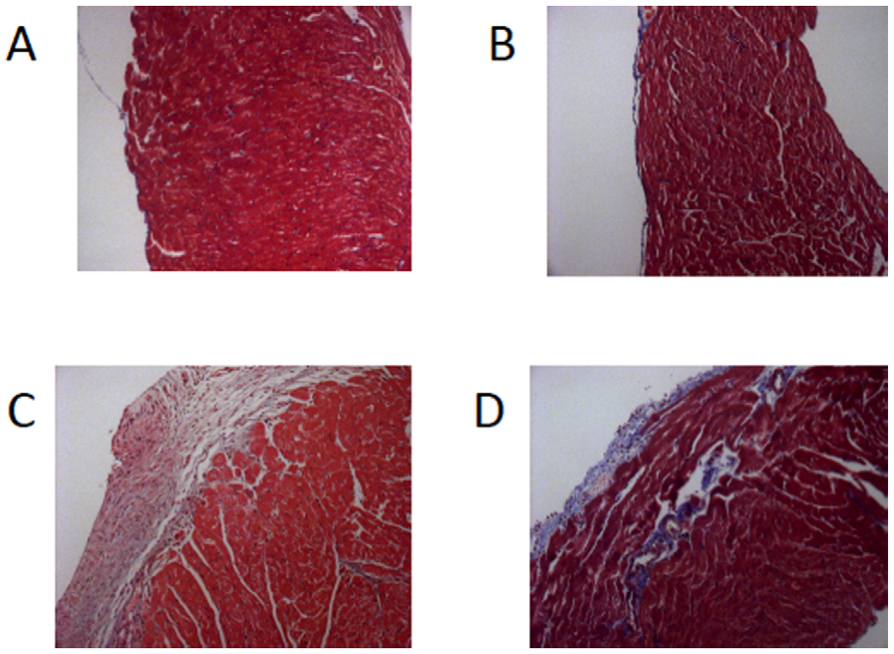
RV fibrosis. Masson's trichrome stain in A) CON.CON B) ACE2.CON 3) CON.PAB 4) ACE2.PAB cardiac tissue. 200× magnification.

**Figure 6:**
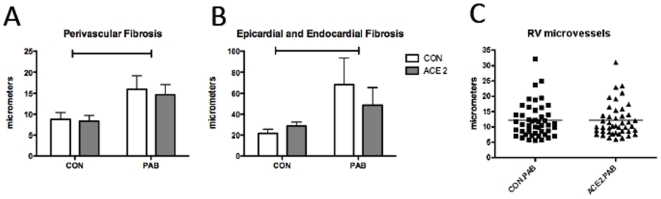
Measurement of RV fibrosis. (A) perivascular, (B) epicardial and endocardial fibrosis in the RV. p<0.05 for PAB effect for large brackets. Number and size of (C) RV microvessels in RV tissue.

We explored potential mechanisms by which rhACE2 diminished RV hypertrophy and improved RV function. There was no significant change in RV fibrosis between PAB and PAB receiving rhACE2 (Figure 5) as measured via epicardial, endocardial, or perivascular fibrosis width (Figure 6). There was also no significant change in RV fibrosis in PAB compared with PAB receiving rhACE2 as evaluated by percentage of collagen area per area cardiac tissue (0.6–0.8% CON.CON, 0.3–1% ACE2.CON, 9.3–11.6% CON.PAB, and 7.9–21% ACE2.PAB). Since ACE2 stimulates endothelial cell tube formation [26], we counted the number of microvessels in the RV. However, the number and size of microvessels was not different between PAB with and without rhACE2 (Figure 6).

### Molecular effects of rhACE2 administration on the RV

**Figure 7:**
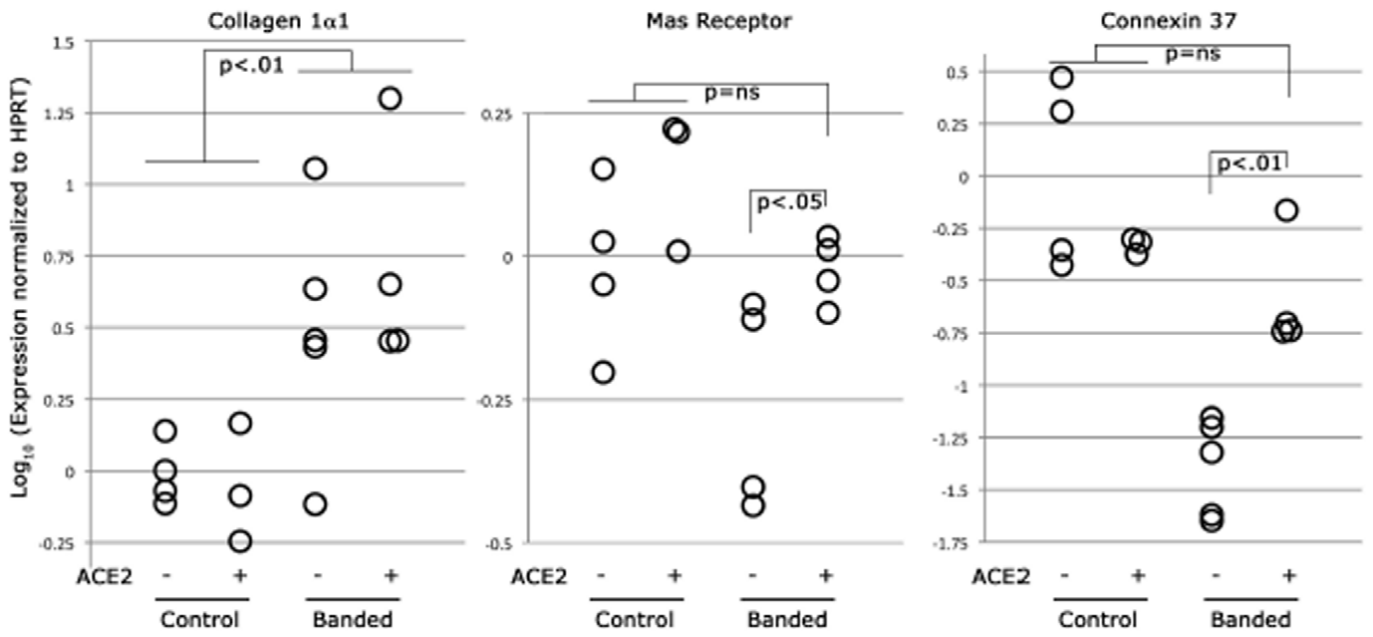
Molecular expression levels in the RV. (A) collagen1α1, (B) Mas receptor, and (C) connexin 37 normalized to HPRT on a log scale. Each open circle represents one mouse.

Expression of collagen1α1, Mas receptor, and connexin 37 were measured in RV tissue to determine whether rhACE2 administration produced molecular effects on the RV (Figure 7). Collagen1α1 expression increased around five-fold in PAB mice (p<0.01) but did not change with rhACE2 administration. In PAB mice without rhACE2, Mas expression was decreased compared with controls; rhACE2 administration increased Mas expression in these mice approximately two-fold (p<0.05). Lastly, connexin 37 was chosen as a marker for cell-cell communication. Connexin 37 is a gap junction protein and is widely distributed in the heart (cardiac endothelial, epithelial and myocardial cells, conotruncal ridges, and atrioventricular cushions) [27]. PAB mice had a 24-fold decrease in connexin 37 expression levels compared with controls; levels were normalized with rhACE2 administration (p<0.01).

## Discussion

In these experiments, rhACE2 administered via osmotic pumps to PAB mice for two weeks prevented load-induced RV systolic and diastolic dysfunction. Importantly, rhACE2 increased power without increasing RV hypertrophy.

**Figure 8:**
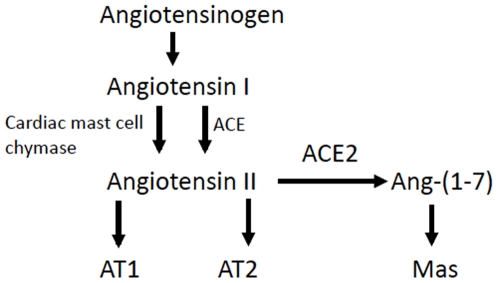
The ACE2 pathway.

Elevated Ang II levels are instrumental in cardiovascular remodeling, and therapies limiting Ang II production or binding are the foundation of cardiovascular therapeutics. Figure 8 depicts a simplified view of the ACE2 pathway; Angiotensin II is converted by ACE2 to Ang-(1-7) which binds to the Mas receptor. Figure 8 also demonstrates that in the heart and vasculature, Ang II is produced by both mast cell chymase and through conventional renin-angiotensin pathway conversion of Ang I to Ang II by angiotensin- converting enzyme (ACE) [28]. Increased mast cell chymase (present in the secretory granules of mast cells) is present in cardiac and vascular tissue in animal models of cardiovascular disease including cardiomyopathy, atherosclerosis, aortic aneurysm, and myocardial infarction [29], [30], [31], [32], [33]. Whereas mast cells are present in the epicardium, endocardium, and occasionally dispersed among cadiomyocytes in our PAB mice, they were only present in the cardiac epicardium and endocardium in our control animals (not shown). In humans, higher chymase mRNA expression and mast cell density is present in failing myocardium compared with controls [34]. Consequently, ACE inhibitors may not effectively suppress Ang II production, and angiotensin receptor blockers, which inhibit Ang II binding, do not increase Ang-(1-7) formation. Therefore, ACE2 is a potential treatment option in RV dysfunction, simultaneously counterbalancing Ang II production in a failing heart and increasing Ang-(1-7) formation.

Generally, ventricular function improves through increasing preload, decreasing afterload, or increasing contractility. ACE2 did not increase preload (RV end diastolic volume), nor did it decrease afterload in our PAB model (the pulmonary vasculature is dilated at baseline). Consequently, RV improvement with ACE2 administration is likely secondary to improved contractility. There was significant increase in EF and stroke volume, and there was a trend towards increased dPdt (maximal initial velocity) with ACE2. Other studies have linked ACE2 with contractility; profound contractile dysfunction occurs after ACE2 gene inactivation in the mouse heart [35]. In an LV ischemic cardiac model, cardiac overexpression of ACE2 via lenti-virus, produced results similar to ours; ACE2 did not change collagen density, cardiomyocyte cross-sectional area, or capillary density in the ischemia model, yet improved LV wall motion and contractility [16].

We evaluated molecular effects of ACE2 on the RV by measuring Mas receptor and connexin expression levels. Review of the literature suggests that ACE2 may improve LV cardiac function through Ang-(1-7) activity at the Mas receptor [36], [37], [38]. In our PAB mice, we demonstrated that Mas expression was decreased and that Mas expression increased with administration of rhACE2. The Mas receptor is a G-protein coupled receptor, but its signaling pathway in the heart is largely unknown. In preliminary data using a transgenic PAH mouse model, we discovered that rhACE2 corrects abnormalities in focal adhesions and adherens junctions in whole lung tissue (unpublished). In the heart, we evaluated intercellular communication through gap junctions which are assembled from membrane-associated proteins called connexins. Connexin 37 levels were lower in PAB mice compared with controls and normalized following rhACE2 administration (Figure 7). Therefore, it is possible that ACE2 improves RV function though connexin regulated contractility.

rhACE2 had minimal effect in control animals on echocardiography and catheterization (administration of rhACE2 to control mice resulted in a small, but statistically significant increase in RV end diastolic pressure and stroke volume). Our study did not examine the underlying mechanisms by which ACE2 may have affected normal RV function, but changes in normal LV function have been observed by other investigators with ACE2 [16] which may represent regulation of this pathway in both normal and abnormal physiology. In our study, there were no detrimental effects on the LV in PAB mice receiving rhACE2. In fact, the trend towards a decrease in LV end diastolic pressure with rhACE2 correlates with a decrease in RVIDd as visualized by echocardiography based on cardiac chamber interdependence. Therefore, in a RV load-stress model of early heart failure, rhACE2 at a rate of 1.8 mg/kg/day appears to be cardioprotective in the RV and safe in the LV.

In human PAH patients, ACE2 may provide therapeutic effects in both the RV and lung. Although the PAB model presented in this manuscript cannot evaluate distal pulmonary vascular disease, success in both monocrotaline and transgenic animal models of PAH support ACE2 as an effective pulmonary vascular treatment. For example, researchers have prevented pulmonary vascular disease in a monocrotaline rat model using XNT, a synthetic activator of ACE2 [39]. In addition, PH was prevented and reversed via gene transfer with ACE2 and Ang-(1-7) lentiviral vectors in monocrotaline models [40], [41]. Our lab administered ACE2 to a transgenic mouse model of PAH and reversed PH (manuscript in review). Therefore, ACE2 may provide synergistic therapy in human PAH patients by improving both RV and lung dysfunction.

Limitations of this study include lack of cardiac conduction evaluation with electrocardiograms. It has been suggested that ACE2 transgene overexpression can cause heart block, ventricular tachycardia, and sudden death [42]. Although Ang-(1-7) benefits failing hearts by increasing the resting potential, action potential, and enhancing conduction velocity and electrical synchronization, anti-arrhythmic properties of Ang-(1-7) are lost at higher levels of Ang-(1-7) administration [43]. Consequently, rhACE2 may have differential effects in the heart depending on dose, and further dosage studies with rhACE2 in heart failure should be performed. Our PAB model evaluates the early phase of heart failure at a time point where the heart is capable of compensation. rhACE2 should also be tested in a chronic pressure-overload model. In the future, we would like to further examine the molecular etiology behind rhACE2 therapy in the RV and whether shorter courses of rhACE2 (3, 5, or 7 days) will result in similar improvement in RV function.

In summary, RV function is a critical determinant of morbidity and mortality in PH [2], [3]. Therapies targeting RV function may provide a new class of drugs for RV failure secondary to PH. This is the first report of targeted RV therapy with rhACE2 in a RV pressure overload model in which preventative rhACE2 therapy in PAB mice improved RV systolic and diastolic dysfunction and diminished stressed-induced RV hypertrophy.

## Methods

### Ethics statement

This study was carried out in accordance with the National Institutes of Health's Public Health Service Policy of Humane Care and Use of Laboratory Animals and in accordance with the Animal Welfare Act. Vanderbilt University Institutional Animal Care and Use Committee approved all procedures (protocol M/08/083). All experiments were performed using appropriate analgesics and anesthetics, and every effort was made to minimize pain and distress.

### PAB and pump insertion

Twenty-seven adult male C57BI/6 mice 10 weeks of age (Jackson Laboratory) were anesthetized with 3% isoflurane, administered intraperitoneal etomidate (20–40 mL/kg), and orotracheally intubated. Animals were mechanically ventilated with isoflurane general anesthesia for PAB or sham surgery. A sternal incision was made using sterile technique, the pericardium was removed, and a partially occlusive tantalum clip was placed around the pulmonary artery (Weck, Research Triangle Park, NC). The animal was then sutured closed and allowed to recover from anesthesia. Mice received post-operative analgesic with a subcutaneous injection of 5 mg/kg of carprofen. Sham treated animals underwent the same procedure without vascular clip placement. On post-op day 2, mice were separated into control or rhACE2 groups: ACE2.PAB n = 10, ACE2.CON n = 5, CON.PAB n = 7, CON.CON n = 5. The rhACE2 group received rhACE2 (Apeiron Biologics, Vienna, Austria) at 1.8 mg/kg/day through micro-osmotic pumps (ALZET 1002). The control group received vehicle alone (100 mM glycine, 150 mM NaCl, 50 uM ZnCl_2_, pH 7.5, Apeiron Biologics). Pumps were placed subcutaneously between the scapula using sterile technique. Mice received analgesic after pump insertion with a subcutaneous injection of 5 mg/kg of carprofen. All assessments were obtained by our technician who was blinded to rhACE2 or placebo.

### Echocardiography

Two-dimensional echocardiography was performed using Vivo 770^©^ High-Resolution Image System (VisualSonics^©^ Toronto, Canada). Echocardiograms were obtained the day prior to sacrifice under isoflurane anesthetic. In the short axis, left and right ventricular end systolic and diastolic diameters and heart rate were recorded in M-mode [44], [45].

### Measurement of RV pressure

Right ventricular systolic pressure (RVSP) was obtained on all but one mouse who died prior to RVSP measurement. Two weeks after receiving rhACE2 or vehicle, mice were anesthetized with 3% isoflurane, administered intraperitoneal etomidate, and orotracheally intubated. Animals were mechanically ventilated with vaporized isoflurane general anesthesia. Mice were positioned supine, ventral side up on a heated operating table. A vertical incision over the abdomen was made and cautery was used to cut the diaphragm and expose the heart. RVSP was directly measured via insertion of a 1.4F Mikro-tip^©^ catheter into a surgically exposed heart. Open-chested catheterization was chosen since this method provides better quality pressure-volume loops than the close-chested technique in our hands. Hemodynamics were continuously recorded with a Millar MPVS-300 unit coupled to a Powerlab 8-SP analog-to-digital converter acquired at 1,000 Hz and captured to a Macintosh G4 (Millar Instruments Houston, TX). After obtaining RV measurements, the catheter was removed and placed into the LV. Mice were sacrificed using a lethal dose of phenobarbital (Schering-Plough). The right and left heart were weighed separately and quick-frozen in liquid nitrogen. Several RV and LV in each group were placed in formalin for histology.

### Histology

Antigen retrieval on cardiac tissue was performed with retrievit-8 (Innogenex, BS-1006-00). Sections were rinsed in DI water and soaked for 20 minutes in 3% H_2_O_2_ (10 ml of H_2_O_2_+90 ml MetOH). Slides were blocked in 10% powerblock (1∶10 dilution universal blocking reagent, Biogenex, HK085-5K) and 0.4% Triton in PBS and placed in a humid chamber for 15 minutes. ACE2 (Abcam, ab59351) primary antibody in the presence of 10% powerblock in 0.4% Triton-x 100 in PBS was added 1∶200 at 4°C overnight. Secondary antibody (Innogenex, AS-2200-16) anti-rabbit Igs, biotinylated goat (mouse/rat-absorbed) was applied and incubated for 20 minutes. Biogenex streptavidin HRP (Innogenex streptavidin enzyme conjugate CJ-1005-25) was added for 20 minutes. Slides were stained with vector NovaRed (SK-4800) and counterstained in Mayers hematoxylin. Hearts were also stained with Masson's trichrome stain and hematoxylin and eosin. Epicardial and endocardial fibrosis was quantified by measuring the width of the three most fibrotic regions per slide. Perivascular fibrosis was measured on the three most fibrotic vessels on each slide. Percentage of collagen per cardiac tissue area was obtained using Nikon NIS-Elements Advanced Research software (Melville, NY). Microvessels were defined as vessels which were not main coronary vessels and were identified using immunofluorescence staining for vWF 1∶100 (DAKO, Denmark, A0082). Microvessels were counted in twenty fields at 400×.

### PCR

RNA was obtained using Qiagen RNeasy Mini Kit (Qiagen, Valencia, CA). First strand cDNA was made using QuantiTect® Reverse Transcription Kit (Qiagen) from 1 µg total RNA. Quantitative real-time PCR was performed using a total reaction volume of 25 µl, containing 5 µl of diluted cDNA, 12.5 µl SYBR Green Supermix (Applied Biosystems Foster City, CA) and 0.03 µl of each oligonucleotide primer (250 µM). PCR was carried out in a StepOnePlus Real Time PCR System (Applied Biosystems) using 40 cycles of 95°C for 15 seconds followed by 60°C for 1 minute with a ten minute 95°C initial soak. Each measurement was made in triplicate and expressed relative to the detection of the standard HPRT. PCR was preformed for the primer sets HPRT, collagen1α1, Mas1, and connexin 37 (Integrated DNA Technologies IDT® Coralville, Iowa).

### Statistics

Statistical tests were two-way ANOVA with post-hoc Bonferroni test performed using GraphPad Prism Plus version 5.0 Software (San Diego, CA). Expression data was log_10_ transformed before statistical analyses. A p value <0.05 was considered significant.
